# Evidence of the impact of systemic inflammation on neuroinflammation from a non-bacterial endotoxin animal model

**DOI:** 10.1186/s12974-018-1163-z

**Published:** 2018-05-17

**Authors:** Chunxia Huang, Michael Garnet Irwin, Gordon Tin Chun Wong, Raymond Chuen Chung Chang

**Affiliations:** 1 Department of Anaesthesiology, LKS Faculty of Medicine, The University of Hong Kong, Room K424, Queen Mary Hospital, Pokfulam, Hong Kong SAR China; 2 Laboratory of Neurodegenerative Diseases, School of Biomedical Sciences, LKS Faculty of Medicine, The University of Hong Kong, Room L4-49, Laboratory Block, Faculty of Medicine Building, 21 Sassoon Road, Pokfulam, Hong Kong SAR China; 3 State Key Laboratory of Brain and Cognitive Sciences, The University of Hong Kong, Pokfulam, Hong Kong SAR China

**Keywords:** Neuroinflammation, Postoperative cognitive dysfunction, Tau proteins, Sevoflurane, Ibuprofen

## Abstract

**Background:**

Systemic inflammation induces neuroinflammation and cellular changes such as tau phosphorylation to impair cognitive function, including learning and memory. This study uses a single model, laparotomy without any pathogen, to characterize these changes and their responses to anti-inflammatory treatment in the intermediate term.

**Methods:**

In a two-part experiment, wild-type C57BL/6N mice (male, 3 month old, 25 ± 2 g) were subjected to sevoflurane anesthesia alone or to a laparotomy. Cognitive performance, systemic and neuroinflammatory responses, and tau phosphorylation were evaluated on postoperative days (POD) 1, 3, and 14. The effect of perioperative ibuprofen intervention (60 mg/kg) on these changes was then assessed.

**Results:**

Mice in the laparotomy group displayed memory impairment up to POD 14 with initial high levels of inflammatory cytokines in the liver, frontal cortex (IL-1β, IL-6, and TNF-α), and hippocampus (IL-1β and IL-8). On POD 14, although most circulating and resident cytokine levels returned to normal, a significant number of microglia and astrocytes remained activated in the frontal cortex and microglia in the hippocampus, as well as abnormal tau phosphorylation in these two brain regions. Perioperative ibuprofen improved cognitive performance, attenuated systemic inflammation and glial activation, and suppressed the abnormal tau phosphorylation both in the frontal cortex and hippocampus.

**Conclusions:**

Our results suggest that (1) cognitive dysfunction is associated with an unbalanced pro-inflammatory and anti-inflammatory response, tauopathy, and gliosis; (2) cognitive dysfunction, gliosis, and tauopathy following laparotomy can persist well beyond the immediate postoperative period; and (3) anti-inflammatory drugs can act rapidly to attenuate inflammatory responses in the brain and negatively modulate neuropathological changes to improve cognition. These findings may have implications for the duration of therapeutic strategies aimed at curtaining cognitive dysfunction following surgery.

**Electronic supplementary material:**

The online version of this article (10.1186/s12974-018-1163-z) contains supplementary material, which is available to authorized users.

## Background

The impact of systemic inflammation on neuroinflammation has received increasing attention. This is because neuroinflammation may trigger the development of Alzheimer-like pathology and even neurodegeneration if long-lasting neuroinflammation occurs [1, 2]. Activation of inflammatory responses in the brain is one of the pathological events leading to neurodegeneration [3]. Experimentally, majority of the animal model utilizes bacterial endotoxin lipopolysaccharides (LPS) or infection using live bacteria to induce systemic inflammation [4, 5]. We have also used live *Escherichia coli* or LPS to study the molecular events of how systemic inflammation triggers neuroinflammation [6, 7]. However, the drawback of using LPS and pathogens is the limited experimental time frame in several days only. Therefore, we adopt an experimental model of laparotomy to simulate the problem of some patients who suffer from cognitive dysfunction after surgery. In this kind of experimental model of laparotomy, many previous studies examined the impact of postsurgical effects up to 7 days only [8–10]. In this study, we had prolonged the examination time frame from 4 h to 2 weeks in order to investigate the early event from gene expression of cytokines to the development of pathology and cognitive dysfunctions.

In this study, we used a single surgical treatment by opening the abdomen to take out the small intestine for massage without any damage on the intestine. Afterward, the intestine was restored back to the abdomen. By using this surgical procedure (laparotomy), we found a transient upregulation of systemic inflammatory cytokines within 4 h. Surprisingly, activation of neuroimmune responses appeared to be long-lasting for 2 weeks as indicated by the morphology of activated microglia and astrocytes, but not the levels of cytokines. Persistent neuroimmune responses can further promote phosphorylation of tau protein, which may lead to the development of tau pathology. Neuroinflammation and tau protein phosphorylation can be further translated into cognitive dysfunctions, as indicated by Y-maze and novel object recognition tests at 2 weeks after laparotomy. More importantly, we can prove that attenuation of systemic inflammation by ibuprofen could reverse all the above impact in the brain. Our results demonstrate that systemic inflammation can exert relatively long-term effects on the brain, which is not because of the long-lasting high levels of pro-inflammatory cytokines. However, once the microglial cells are activated, they may play the major role in sustaining neuroimmune responses, resulting in cognitive dysfunctions. Our study may reshape clinical practice of targeting systemic immune responses in order to minimize the damage to the brain after surgery.

## Methods

### Animals

Three-month-old male C57BL/6N mice (25 ± 2 g) were obtained from the Laboratory Animal Unit (LAU) of The University of Hong Kong and housed according to Association for Assessment and Accreditation of Laboratory Animal Care (AAALAC). All experimental protocols and animal handling procedures were approved by the Faculty Committee on the Use of Live Animals in Teaching and Research of the university (CULATR, Ref. No. 3437-14). The mice were housed in a temperature-controlled room at 20–22 °C, humidity of 50 ± 10%, and were kept on a 12/12-h light/dark cycle. All animals had access to food and water ad libitum, and they underwent an acclimatization period for 1 week before being employed in the experiment. All behavioral tests have been performed from 09:00 to 12:00 A.M. during the light phase.

### Experimental protocols

In the first experiment, mice were randomly divided into the following groups: control (CON), sevoflurane anesthesia (SEVO), and laparotomy under sevoflurane anesthesia (LAP). In the second experiment, mice were randomly assigned to undergo laparotomy with (LAP+Ibu) or without (LAP) perioperative ibuprofen administration. Ibuprofen (Sigma-Aldrich, USA) (60 mgkg^−1^ day^−1^) [11] was administrated orally in drinking water for 14 days, with the first dosage given by gavage 1 hour before the laparotomy. To evaluate biochemical and histological characterization of inflammation induced by laparotomy, we measured mRNA expression and protein levels of inflammatory cytokines in the liver, brain, and plasma, as well as the activation of glial cells in the brain. Cognitive performance was evaluated by serial behavioral tests including open field test, novel object recognition test, and Y-maze test. Tau protein phosphorylation and related signaling pathways were comparatively determined on postoperative day 14 by Western blot analysis.

### Surgical and anesthetic procedures

Anesthesia was induced with sevoflurane (Sevorane™, Abbott, Switzerland) at 5% and maintained at 3% sevoflurane using a rodent inhalation anesthesia apparatus (Harvard Apparatus, USA) with a fresh gas flow of 800 mlmin^−1^. We modified our surgical procedure from those previous studies [12, 13]. For the surgical procedure, a 2.5-cm longitudinal midline incision was made in the abdomen, and then approximately 10 cm of the intestine was exteriorized and vigorously rubbed for 30 s. The bowel loops remained outside the abdominal cavity for 1 min and then replaced into the abdominal cavity. Sterile gut sutures (4-0, PS-2; Ethicon, USA) were used to suture the peritoneal lining and abdominal muscle in two layers and the skin. The entire procedure was completed within 15 min with monitoring of the rhythm and frequency of respiration and the color of animals’ paw on the heating pad. Mice from the anesthesia only group were subjected to 15 min of sevoflurane anesthesia at the same concentrations and gas flow.

### Von Frey filament test

Von Frey filament (VFF) test was performed at postoperative 24 h. VFFs of six different calibers (0.4, 0.6, 1.4, 4, 6, 10 g; North Coast Medical, Morgan Hill, CA) were applied to the abdomen in ascending order three times, each for 1 to 2 s with a 10-s interval between applications. The areas designated for stimulation were 1 cm from the longitudinal midline. A positive response consisted of the rat raising its belly (withdrawal response).

### Open field test

OFT is a classic experimental tool to evaluate general locomotor activity and anxiety in rodents based on their innate tendency to avoid open spaces [14]. During the spontaneous exploration period in an enclosed gridded arena, ambulation was measured in the first 5 min, defined as the total grid line crossing. Total exploration time in the central area was also recorded as the parameter for anxiety.

### Novel object recognition test

Novel object recognition (NOR) task evaluates the rodents’ ability to recognize a novel object in a controlled environment [15]. Twenty-four hours after habituated in the open-field arena in the absence of objects, the mice were placed in the same arena containing two identical sample objects (A + A). After a retention interval (24 h), the animal was returned to the arena with two objects, one is identical to the sample and the other is novel (A + B). The discrimination index was used to evaluate the recognition memory as the ratio of the exploration time of one object to two objects.

### Y-maze training and test

The modified Y-maze test is used to assess hippocampal-dependent special learning capacity [8]. After habituation for assessing spontaneous alternating behavior, each mouse was placed in one of the black compartments, and electric shocks (2 Hz, 10 s, 40 ± 5 V) were applied until it entered the shock-free compartment and stayed there for 30 s. This was recorded as a correct choice. Successful training was made with continuous nine correct choices. For the testing trial, each mouse was tested ten times following the same procedures as in the training trial. The number of incorrect choices as well as the time taken to enter the shock-free compartment (latency) was recorded.

### Real-time quantitative reverse-transcription polymerase chain reaction for mRNA

Total RNA from tissues was isolated using TRI Reagent® (MRC, Cincinnati, USA). Only the isolated RNA samples with an OD260/280 ratio > 1.8 and OD260/230 ratio < 2.0 were used for analysis. After further purification with Ambion® DNA-free™ DNA Removal Kit (Invitrogen, USA) and reverse transcription using PrimeScript™ Master Mix Kit (TAKARA, Japan), PCR was performed using StepOnePlus™ Real-Time PCR system (Applied Biosystems, USA) with the SYBR®Premix Ex Taq™ II Kit (TAKARA, Japan). The amplification conditions were 95 °C for 20 s, followed by 40 cycles of denaturation at 95 °C (15 s), extension at different gene-specific annealing temperature as described in Table 1, and data capture at 72 °C (30 s). The relative levels of cytokines were normalized to the endogenous reference glyceraldehyde-3-phosphate dehydrogenase (GAPDH) following the 2^-ΔΔCt^ method.

**Table 1:** PCR conditions for inflammatory cytokines

Gene	Primer sequences	Annealing temperature (°C)
Interleukin-1-β (IL-1β)	F: 5′-CCTCCTTGCCTCTGATGG-3′ R: 5′-AGTGCTGCCTAATGTCCC-3′	60
Tumor necrosis factor (TNF-α)	F: 5′-CCCCAGTCTGTATCCTTCT-3′ R: 5′-ACTGTCCCAGCATCTTGT-3′	59
Interleukin-6 (IL-6)	F: 5′-GGCAATTCTGATTGTATG-3′ R: 5′-CTCTGGCTTTGTCTTTCT-3′	56
Interleukin-8 (IL-8)	F: 5′-TGCCGTGACCTCAAGATGTGCC-3′ R: 5′-CATCCACAAGCGTGCTGTAGGTG-3′	60
Interleukin-10 (IL-10)	F: 5′-CCAAGCCTTATCGGAAATGA-3′ R: 5′-TTCTCACCCAGGGAATTCAA-3′	60
Glyceraldehye-3-phosphate dehydrogenase (GAPDH)	F: 5′-ATTCAACGGCACAGTCAA-3′ R: 5′-CTCGCTCCTGGAAGATGG-3′	56

### SDS-PAGE and Western blot analysis

Mice were sacrificed by CO_2_ asphyxiation following all behavioral tests, in accordance with the guidelines of the American Veterinary Medical Association. Blood was transcardially collected and then centrifuged at 1400*g* for 14 min for plasma separation. After transcardial perfusion with cold 0.9% saline, the right hemispheres were fixed with 4% paraformaldehyde for 72 h and then dehydrated in serial ethanol and embedded in paraffin for immunofluorescence. The hippocampal and frontal cortical tissues were dissected from the left hemisphere for Western blotting. Total proteins were collected and subjected to 10% polyacrylamide gel electrophoresis as described previously [16]. After blocking with 5% non-fat dry milk, the membranes were incubated overnight at 4 °C with specific primary antibodies (Table 2). Horseradish peroxidase-conjugated secondary antibodies (DAKO, Denmark) were then used. The immunoreactive band signal intensity was subsequently visualized by chemiluminescence (ECL or ECL-plus, Amersham GE Healthcare, UK). All immunoblots were normalized for gel loading with β-actin, GAPDH, or α-Tubulin antibodies. The intensities of chemiluminescent bands were measured using Image-J software (National Institutes of Health, USA).

**Table 2:** Primary antibodies used in Western blot analysis

Phosphor peptide/kinase	Dilution	Resource	Catalog no.
AT8 (pSer^202^/Thr^205^)	1:1000	Thermo Fisher Scientific, USA	MN1020
AT180 (pThr^231^/Ser^235^)	1:1000	Thermo Fisher Scientific, USA	MN1040
pS404 (pSer^404^)	1:3000	Biosource, USA	44-758G
Total tau (polyclonal rabbit anti-human tau)	1:30,000	DAKO, Denmark	A0024
Jak2	1:1000	Cell Signaling Technology	3230S
phospho-Jak2 (Tyr^1007/1008^)	1:1000	Cell Signaling Technology	3771S
Stat3	1:1000	Cell Signaling Technology	9139S
Phospho-Stat3 (Tyr^705^)	1:1000	Cell Signaling Technology	9131S
Glycogen synthase kinase-3β (GSK-3β)	1:1000	Cell Signaling Technology	9315S
Phospho-GSK-3β (Ser^9^)	1:1000	Cell Signaling Technology	9336S
Extracellular signal-regulated kinase (ERK) 1/2	1:3000	Cell Signaling Technology	9102
Phospho-ERK1/2 (Thr^202^/Tyr^204^)	1:3000	Cell Signaling Technology	9101S
Stress-activated protein kinases (SAPK)/c-Jun N-terminal kinase (JNK)	1:3000	Cell Signaling Technology	9258S
Phospho-SAPK-JNK (Thr^183^/Tyr^185^)	1:1000	Cell Signaling Technology	9251S
PP2A-C	1:3000	Millipore, USA	05-421
Phospho-PP2A (Tyr^307^)	1:3000	Epitomics	1155-1
β-actin	1:30,000	Sigma-Aldrich	A5441
Glyceraldehye-3-phosphate dehydrogenase (GAPDH)	1:3000	Sigma-Aldrich	G8795
α-Tubulin	1:40,000	Sigma-Aldrich	T9026

### Immunofluorescence staining and confocal microscopy

In brief, before antigen retrieval and blocking, 6-μm-thick coronal sections (frontal cortex—from 2.46 to 1.98 mm anterior to bregma; hippocampus—from − 1.46 to − 2.46 mm posterior to bregma) were deparaffinated and rehydrated. Then the brain sections were incubated at 4 °C overnight with the following primary antibodies: Iba1 (1:400, Wako, Japan) and glial fibrillary acidic protein (GFAP) (1:400, Millipore, USA). Alexa Fluor 568 goat anti-mouse or Alexa Fluor 488 goat anti-rabbit secondary antibodies (1:400, Invitrogen, USA) were used. Sections were co-stained with 5 μM 4′-6-diamidino-2-phenylindole (DAPI) to identify the cell nucleus. Immunolabeled tissues were observed under a Carl Zeiss LSM 700 confocal microscope (× 5, × 20, and × 40 oil immersion objectives) at 1024 × 1024 resolution equipped with ZEN light software. *Z*-stack images were acquired and exported by using the Image-J software. All qualitative analyses were performed on at least four images acquired from at least four serial sections per animal from at least three independent experiments.

### Milliplex cytokine assays

Protein levels of IL-1β, IL-6, MIP-2, TNF-α, and IL-10 in whole protein lysates or plasma were measured using a customized Milliplex Mouse Cytokine Immunoassay Kit (2620525)/MILLIPLEX MAP Mouse Cytokine/Chemokine Magnetic Bead Panel, MCYTOMAG-70K with Analyzer 3.1 Luminex 200 machine (Millipore, USA). Data were analyzed on corresponding software according to the manufacturer’s instructions.

### Statistical analysis

Data are represented as the mean ± SEM and analyzed by using the statistic software GraphPad Prism (version 6.0; Graph Pad Software Inc., USA). A one-way ANOVA followed by Bonferroni’s post hoc tests was used to assess differences among CON, SEVO, and LAP groups. Unpaired two-tailed Student’s *t* test was used to compare the differences between LAP and LAP+Ibu groups. Normality of the data and homogeneity of group variances were assessed using the D’Agostino-Pearson omnibus normality test, Shapiro-Wilk normality test, and Kolmogorov-Smirnov test, respectively. Statistical significance was determined if *p* < 0.05.

## Results

### Activation of immune responses in systemic and central nervous system after laparotomy

**Fig. 1:**
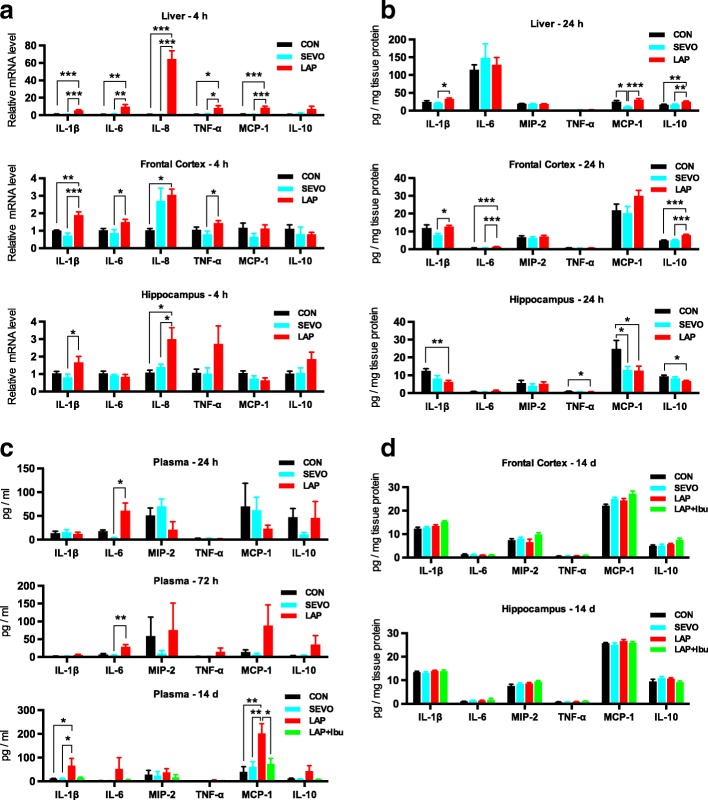
Laparotomy induced acute and persistent peripheral inflammation and neuroinflammation. **a** Relative mRNA levels of pro- and anti-inflammatory cytokines in the liver, frontal cortex, and hippocampus showed a pro-inflammatory state at 4 h after laparotomy. **b** Cytokines protein expressions measured by MILLIPLEX assay of whole tissue lysates at 24 h for the brain and liver. **a**, **b** *n* = 7–8; *p < 0.05, **p < 0.01, ***p < 0.001. **c** Systemic inflammation was determined by the increases of pro-inflammatory cytokines in the plasma on POD 1, 3, and 14. At 24 and 72 h, *n* = 7–8; at 14d, *n* = 8–12; *p < 0.05, **p < 0.01. For IL-1β, LAP+Ibu vs. LAP, *p* = 0.0685. **d** On postoperative day (POD) 14, similar cytokine concentration in the brain after sevoflurane anesthesia or laparotomy under sevoflurane anesthesia with or without ibuprofen consumption (*n* = 8–12)

The presence of peripheral inflammation was demonstrated by high levels of mRNA for pro-inflammatory cytokines in the liver at 4 h, while the presence of neuroinflammation was indicated by the increases in mRNAs for interleukin-1β (IL-1β), IL-6, and tumor necrosis factor (TNF-α) in the frontal cortex (Fig. 1a; 163.82, 71.73, 80.87% increase compared to SEVO group; *p* < 0.0001, *p* = 0.0351, *p* = 0.036) and IL-1β and IL-8 in the hippocampus (Fig. 1a; 106.88, 113.89% increase compared to SEVO group; *p* = 0.0452, *p* = 0.037). At 24 h, elevations of IL-1β and IL-10 levels were found in the liver (Fig. 1b; 56.35, 47.34% increase compared to SEVO group; *p* = 0.0249, *p* = 0.0033) and in the frontal cortex (Fig. 1b; 59.32, 58.4% increase compared to SEVO group; *p* = 0.0252, *p* < 0.0001). In the laparotomy group, level of circulating cytokines such as IL-6 remained elevated up to 72 h (Fig. 1c; 567.34% increase compared to SEVO group; *p* = 0.0038), IL-1β and MCP-1 up to 14 days (Fig. 1c; 527.65 and 366.90% increase compared to SEVO group; *p* = 0.0334, *p* = 0.0081). However, by 14 days of postoperation, there was no longer any increase in expression of any cytokines in the brain, suggesting a resolution of the neuroinflammation (Fig. 1d).

**Fig. 2:**
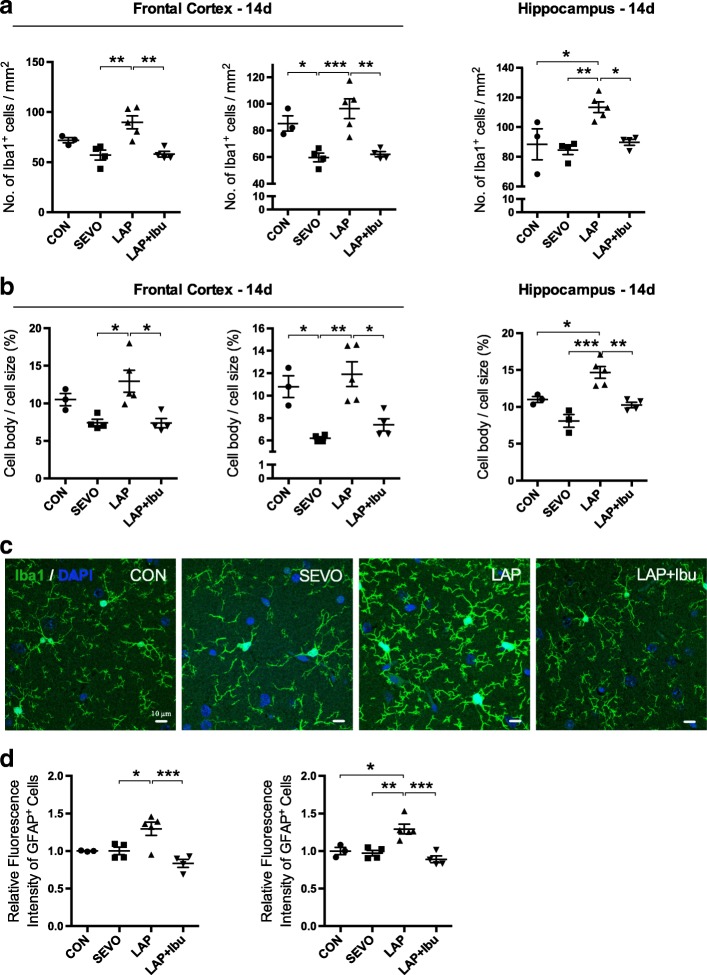
Activation of glia in the brain from surgical mice. **a** In the motor cortex, the number of Iba1^+^ microglia was quantified by using Kruskal-Wallis test with Dunn’s correction, Kruskal-Wallis statistic = 15.11, LAP vs. SEVO, **p = 0.0051; LAP+Ibu vs. LAP, **p = 0.0077. In the sensory cortex, the cell count of Iba1^+^ microglia was analyzed by using Kruskal-Wallis test with Dunn’s correction, Kruskal-Wallis statistic = 21.76, *p = 0.03, **p = 0.0023, and ***p = 0.0008. In the hippocampus, the number of Iba1^+^ microglia was quantified by using one-way ANOVA (*n* = 3–5, *F* = 5.492; LAP vs. CON, *p = 0.0218; LAP vs. SEVO, **p = 0.0041; LAP+Ibu vs. LAP, *p = 0.0182). Dots in the graphs represent the mean value of the four brain sections per mouse. **b** The percentage of cell body to the total cell size of Iba1^+^ microglia was quantified by using one-way ANOVA (*n* = 3–5). In the motor cortex, *F* = 7.146; LAP vs. SEVO, *p = 0.0134; LAP+Ibu vs. LAP, *p = 0.0125. In the sensory cortex, *F* = 10.99; LAP vs. CON, *p = 0.0226; LAP vs. SEVO, *p = 0.0017; LAP+Ibu vs. LAP, *p = 0.0107. In the hippocampus, *F* = 16.99; LAP vs. CON, *p = 0.0193; LAP vs. SEVO, **p = 0.0002; LAP+Ibu vs. LAP, **p = 0.0029. Dots in the graphs represent the mean value of the four brain sections per mouse. **c** Representative confocal microphotographs presented the activation of Iba1^+^ microglia in the hippocampus. **d** The activation of GFAP^+^ astrocyte was quantified using one-way ANOVA. In the motor cortex (left), *F* = 8.969, *p = 0.0452, and **p = 0.0018. In the sensory cortex (right), *F* = 12.52, *p = 0.0165, **p = 0.0045, and ***p = 0.0007

Up to 14 days following laparotomy, Iba1^+^ microglia were increased in the whole frontal cortex (Fig. 2a; 59.52% increase compared to SEVO group, *p* = 0.0216) and its subregions, as well as the hippocampus (Fig. 2b; 28.31% increase compared to SEVO group, *p* = 0.0094). Furthermore, the morphology of increased cell bodies was pronounced presented in the laparotomy group (Fig. 2c). This is compared with the predominately resting microglia seen in the control and sevoflurane groups. Notable GFAP^+^ astrocytes were also observed in the postsurgical frontal cortex (Fig. 2d; 29.85% increase compared to SEVO group, *p* = 0.0359) but were absent in the hippocampus (see Additional file 1: Figure S1 and S2).

### Tau protein phosphorylation induced by laparotomy

**Fig. 3:**
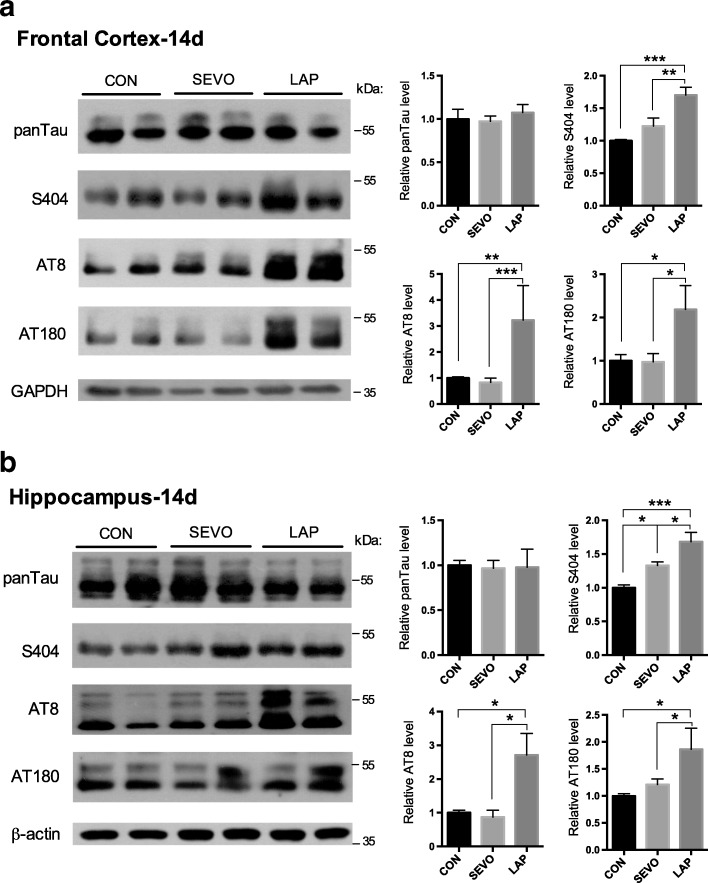
Tau protein phosphorylation following sevoflurane anesthesia or laparotomy on POD 14. Tau phosphorylation levels at different phosphorylation sites recognized by antibodies S404 (Ser^404^), AT8 (Ser^202^/Thr^205^), AT180 (Thr^231^/Ser^235^), and panTau in the **a** frontal cortex and **b** hippocampus (*n* = 8, *p < 0.05, **p < 0.01, ***p < 0.001)

**Fig. 4:**
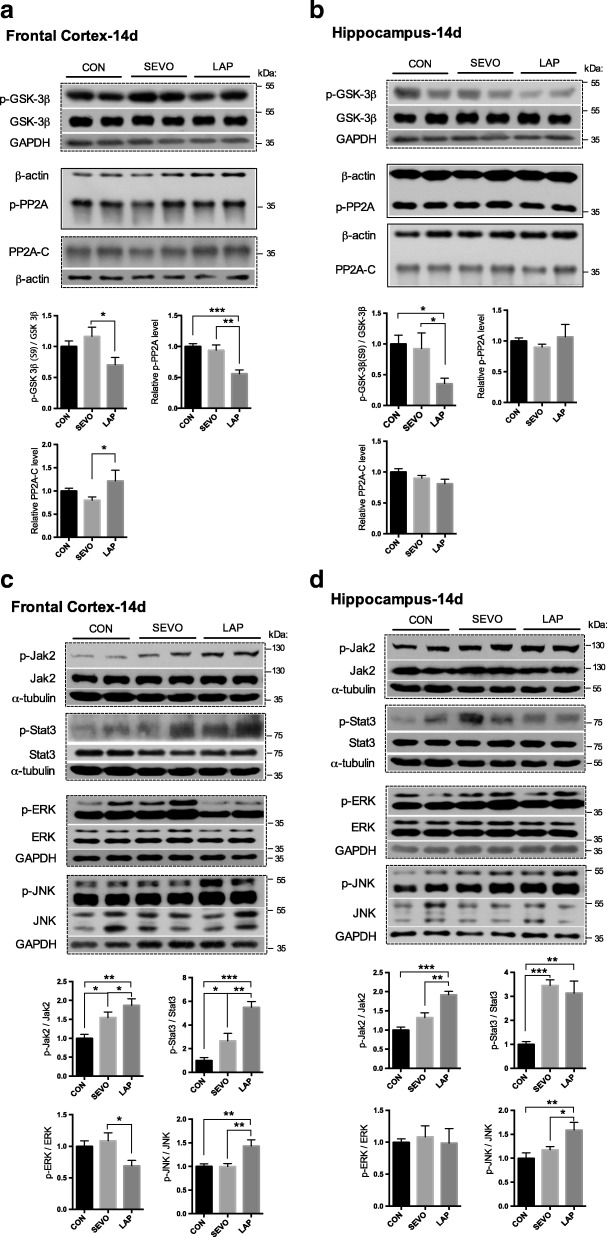
Tau protein phosphorylation-related signaling pathways following sevoflurane anesthesia or laparotomy on POD 14. **a** & **b** Differential changes of GSK3β and phosphatase (PP2A) in the frontal cortex (**a**) and hippocampus (**b**). Relative levels of p-GSK3β (Ser^9^)/GSK3β and p-PP2A (Tyr^307^) in Western blot analysis. **c** & **d** Relative levels of p-Jak2 (Tyr^1007/1008^)/Jak2, p-Stat3 (Tyr^705^)/Stat3, p-ERK/ERK, and p-JNK/JNK in the frontal cortex (**a**) and hippocampus (**d**) were evaluated by using Western blot analysis. For each panel, *n* = 8, *p < 0.05, **p < 0.01, ***p < 0.001

The predictive role of abnormal phosphorylation of tau protein in the pathogenesis of cognitive impairment and neuronal apoptosis in human diseases or postoperative changes, such as Alzheimer’s disease (AD), is well demonstrated by a number of studies [17, 18]. The phosphorylation sites of tau tested were selected based on our preliminary experiments. On postoperative day (POD) 14, the three sites tested had an increase in phosphorylation after laparotomy both for the frontal cortex and hippocampus (S404, AT8, and AT180) (Fig. 3a, b). Tau protein phosphorylation status is dependent upon the balance between the activity of the kinases and phosphatases (Fig. 4). In the frontal cortex, the activation of GSK3β and the inhibition of PP2A may provide evidence for the elevated tau phosphorylation induced by laparotomy (Fig. 4a). While in the hippocampus, laparotomy enhanced the activation of GSK3β without affecting the activity of PP2A (Fig. 4b).

**Fig. 5:**
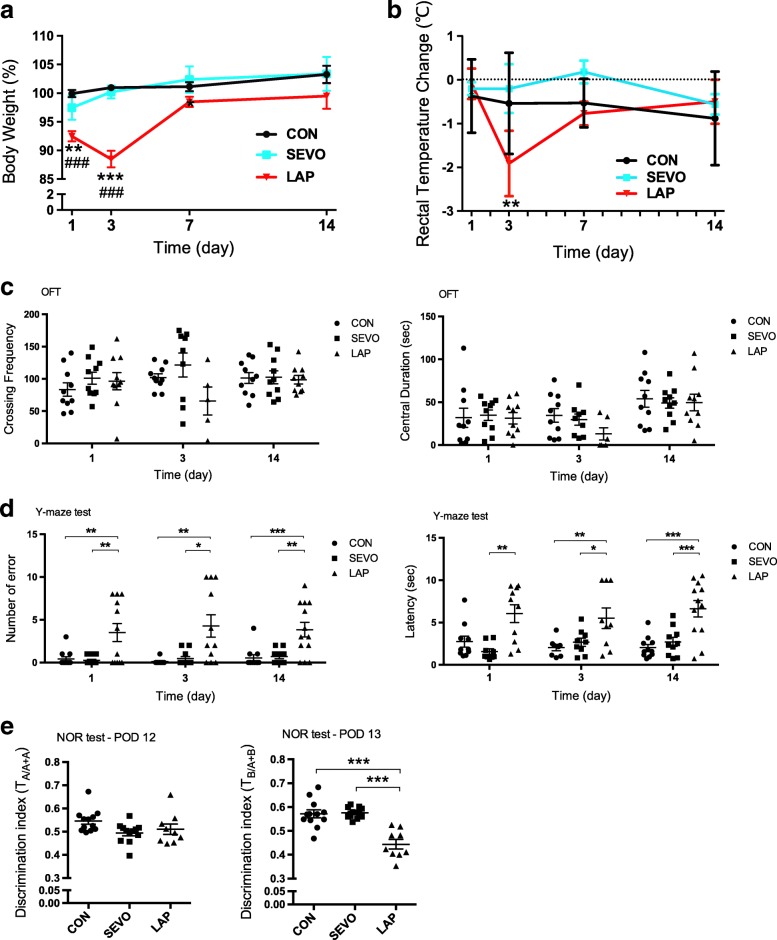
Persistent cognitive impairment during postoperative period. **a** Body weights as percentage of baseline were analyzed using a two-way ANOVA followed by Bonferroni’s post hoc test (*F*_2,103_ = 42.75; LAP vs. SEVO, *p = 0.0445, **p = 0.0059, ***p < 0.0001; LAP vs. CON, ^###^*p* < 0.0001; on POD 14, LAP vs. SEVO, *p* = 0.0531). **b** Rectal temperature was analyzed by two-way ANOVA followed by Bonferroni’s post hoc test (**p = 0.0071, *F* = 2.588). **c** In the open filed test, locomotor activity and anxiety indicated by grid crossing frequency and central exploration time respectively on POD 1, 3, and 14. **d** In the Y-maze test, longer escape latency and greater error number showed working memory deficits on POD 1, 3, and 14. **e** In the novel object recognition (NOR) test, discrimination index (DI) was the ratio of exploration time of one object to two objects, old object (A) to (A + A) on POD 12, or new object (B) to (A + B) on POD 13. There was no object and location preference during the familiarization session. **a**–**e** *n* = 9–12, *p < 0.05, **p < 0.01, ***p < 0.001

On POD 14 following laparotomy, the activities of JAK/STAT3 and JNK were upregulated through phosphorylation in both the frontal cortex and hippocampus, indicating the persistent cellular stress. On the other hand, laparotomy negatively modulated the activity of cell survival-related kinase ERK evidenced by the reduction of phosphorylated ERK in the frontal cortex (Fig. 4c, d).

### Cognitive impairment following laparotomy

Significant weight loss was observed in mice after laparotomy at different postoperative time points that were evident from the first 24 h and remained different at POD 7 with the greatest decline seen at POD 3 (Fig. 5a). The rectal temperature was also changed after laparotomy, dropping about 1 °C at POD 3, but thereafter returned to normal (Fig. 5b).

There were no major variations in locomotor activity (grid crossing) and exploratory behaviors (central duration) in the open field test between the SEVO and LAP groups during the postoperative period (Fig. 5c). However, following laparotomy, a greater number of errors (Fig. 5d; 3.5 ± 3.7, 4.3 ± 4.3, 3.4 ± 2.8; *p* = 0.0028, *p* = 0.0321, *p* = 0.009) was seen in the Y-maze test for all time points, as well as a longer latency (Fig. 5d; 6.05 ± 3.30, 5.51 ± 3.66, 6.58 ± 3.46 s; *p* = 0.0014, *p* = 0.0151, *p* = 0.0003), indicating that memory impairment is present soon after and persisted into the postoperative period. And the impairment of recognition memory induced by laparotomy was indicated by a lower discrimination index in the NOR test (Fig. 5e; 0.44 ± 0.06 in the LAP group vs. 0.58 ± 0.02 in the SEVO group; *p* < 0.0001). There was no difference in the objective measurement of pain to account for the observed behavorial results (see Additional file 1: Table S1).

### Effects of ibuprofen on inflammation, tau protein phosphorylation, and cognitive performance

Perioperative ibuprofen consumption was associated with a downward trend in the levels of circulating cytokines but only MCP-1 reached statistical significance, indicating some anti-inflammatory effects of the drug (Fig. 1c). There was also attenuation of the microgliosis and astrogliosis triggered by laparotomy in the frontal cortex (Fig. 2a, 35.47, 35.54% reduction compared to LAP group; *p* = 0.0216, *p* = 0.0039). In the hippocampus, ibuprofen suppressed the activation of microglia (Fig. 2b, 16.30% reduction compared to LAP group, *p* = 0.0478).

**Fig. 6:**
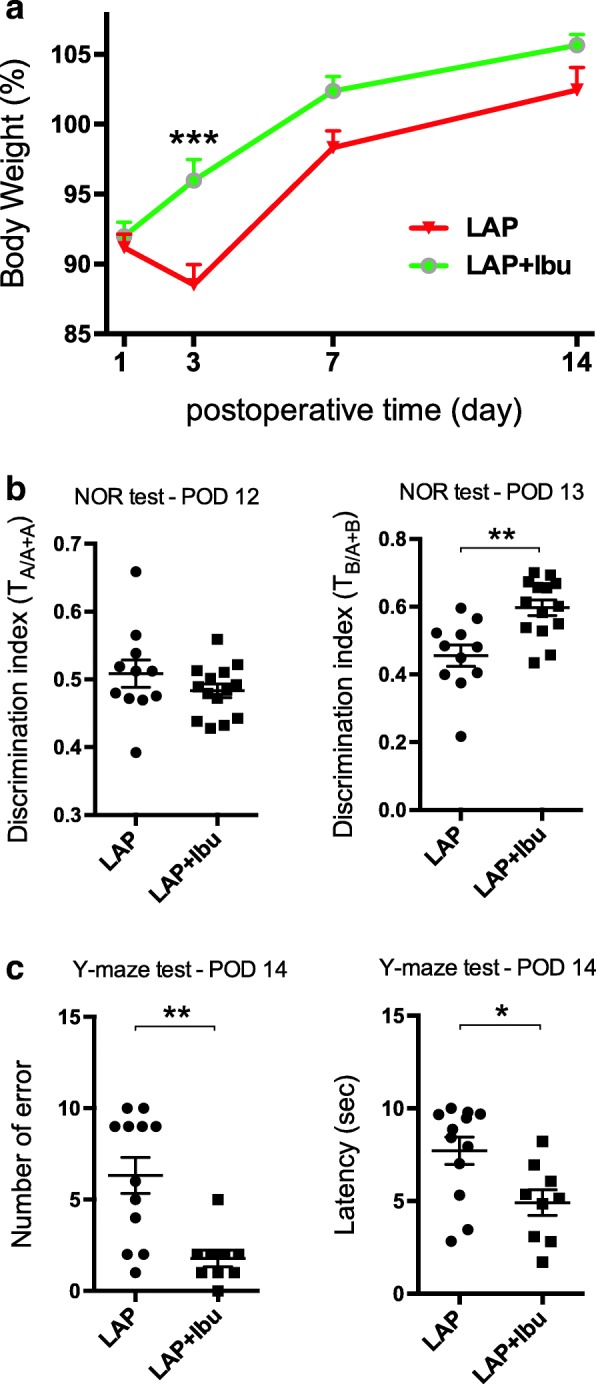
Perioperative ibuprofen intervention prevented cognitive dysfunction. **a** Percentage body weight changes with and without ibuprofen administration. Two-way ANOVA followed by Bonferroni’s post hoc test (***p = 0.0001, *F*_1,108_ = 20.06). **b** Reduction in DI decline in the NOR test after laparotomy with ibuprofen treatment. ***p = 0.0002, *t* = 4.301; Student’s two-tailed *t* test. **c** Reduced error number, Mann-Whitney test, Mann-Whitney *U* statistic = 14, **p = 0.0029, and shorter latency in the Y-maze test, *p = 0.0142, *t* = 2.698; Student’s two-tailed *t* test. **a**–**c** *n* = 11 in LAP group and *n* = 14 in LAP+Ibu group

The ibuprofen-consuming mice displayed less body weight loss (Fig. 6a), less impairment in recognition memory (Fig. 6b; 0.60 ± 0.09 in LAP+Ibu vs. 0.46 ± 0.11 in LAP, *p* = 0.0012), and lower memory deficits (Fig. 6c; 71.93% decrease in error number compared to LAP group, *p* = 0.0029; 33.33% decrease in latency compared to LAP group, *p* = 0.0142).

**Fig. 7:**
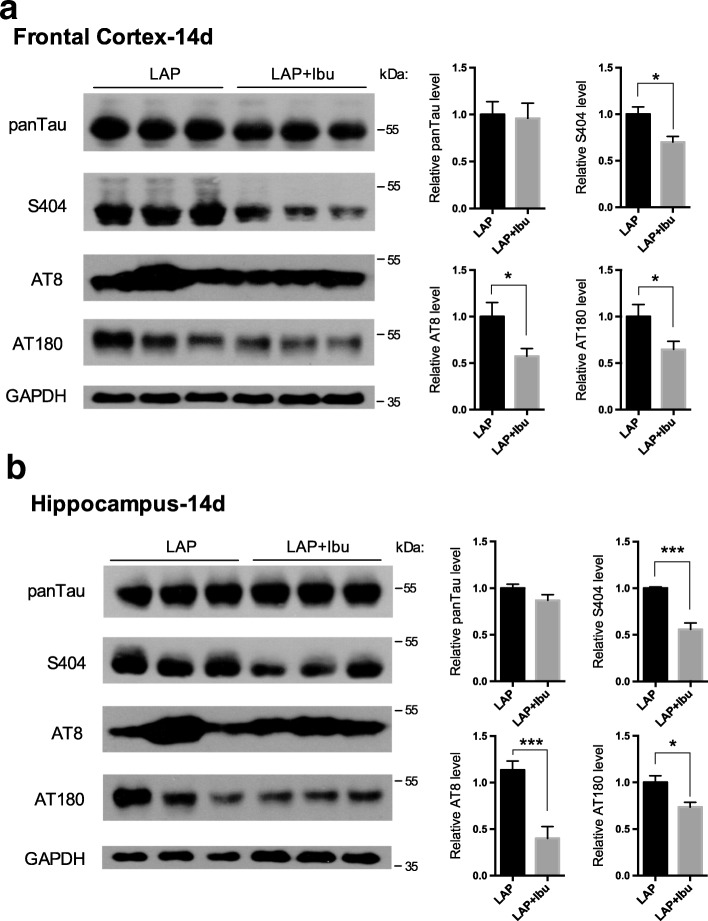
Perioperative ibuprofen intervention prevented tau protein hyperphosphorylation induced by laparotomy. Ibuprofen attenuated tau phosphorylation without affecting total tau in the **a** frontal cortex and **b** hippocampus. For each panel, *n* = 6, *p < 0.05, **p < 0.01, ***p < 0.001

**Fig. 8:**
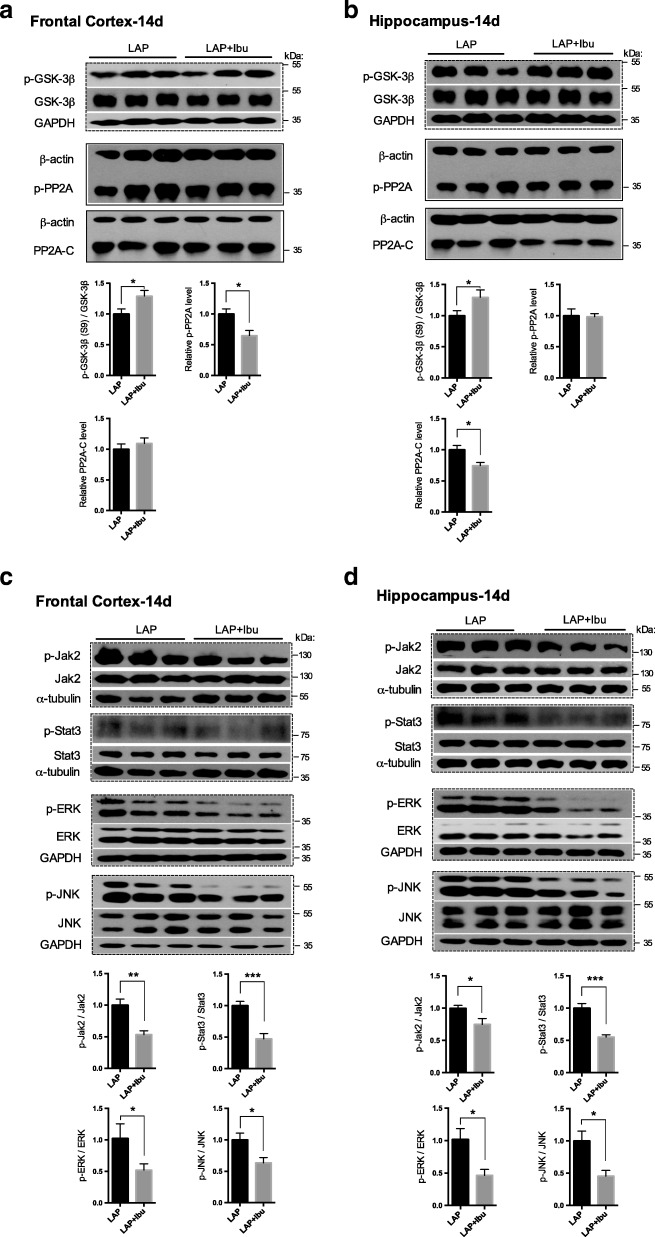
Perioperative ibuprofen intervention inhibited the activation of tau phosphorylation related kinases and cellular stress signaling pathways induced by laparotomy. **a** & **b** Effect of ibuprofen on GSK3β and phosphatase (PP2A) in the frontal cortex (**a**) and hippocampus (**b**), **c** & **d** stress (Jak2/Stat3 and JNK) and cell survival (ERK) signaling pathways in the frontal cortex (**c**) and hippocampus (**d**). For each panel, *n* = 6, *p < 0.05, **p < 0.01, ***p < 0.001

Concurrently, there were decreases of tau protein phosphorylation in the frontal cortex and the hippocampus (S404, AT8, and AT180) (Fig. 7a, b). The increase in phosphorylation of GSK3β at Ser9 as an important inhibitory epitope of the kinase for tau phosphorylation may contribute to the low levels of phosphorylated tau by ibuprofen (Fig. 8a, b).

After ibuprofen administration, stress-related signaling pathway JAK/STAT3 and kinase JNK were less active as well as cell survival-related kinase ERK (Fig. 8a, b). It indicated that stress signaling pathways and major tau kinase GSK3β may be the major mechanisms mediating the neuroprotective effect of ibuprofen against tau phosphorylation induced by laparotomy. The cell survival-related kinases and tau phosphatase were relatively less prominent.

## Discussion

In this study, we used a clinically relevant experimental surgical model to demonstrate cognitive dysfunction accompany changes that bear resemblance to pathological processes that underlie more indolent neurodegenerative disorders. Though some of our findings have been shown in other separate studies, few investigators have incorporated them in a single model and examining the changes at a prolonged postoperative time point. Of particular note, we have demonstrated that there is evidence of persistent gliosis 2 weeks postoperatively accompanying cognitive deficits, and such changes may be attenuated by sustained anti-inflammatory treatment.

In this model, laparotomy but not sevoflurane alone induced peripheral inflammation and neuroinflammation, as well as tau phosphorylation. The inflammatory response occurred very early following surgery, but the cytokine levels in the plasma and the brain essentially resolved by day 14 with the exception of plasma IL-1β and MCP-1. However, there remained a greater number of microglial cells and more in the activated morphology both in the hippocampus and frontal cortex and a similar picture for astrocytes in the frontal cortex. These changes were accompanied by deficits in recognition memory and hippocampus-dependent working memory with no significant difference in motor activity. A sustained anti-inflammatory treatment with ibuprofen decreased tau phosphorylation and improved cognition via anti-inflammatory actions.

It has been shown that inflammatory processes may contribute to the development of neurodegenerative changes, even before any changes of tau [19]. Peripheral inflammation is associated with the production of both pro- and anti-inflammatory cytokines, which activate glial cells and attribute to the progression of neurodegenerative diseases [20]. More recently [13, 21] and now in this study, the role of inflammation has been shown for postoperative cognitive dysfunction. In Hovens’s studies by using another abdominal surgery model (ischemia-reperfusion of the upper mesenteric artery), significantly increased IL-1β and microgliosis are observed at 7 days after surgery, associated with impaired special learning and memory. However, persistent activation of microglia and memory deficits disappeared in postoperative 2 or 3 weeks in young rats [21] while continued to postoperative day 14 [22] or even postoperative 6 weeks [23] in aged rats. In our study, we found persistent microgliosis and cognitive deficits (special memory and object recognition) on postoperative day 14 in young mice (3 months old). And the glial cells may remain activated even after the resolution of cytokine elevation. The acute and constant inflammatory responses in the brain may contribute to the persistent cognitive dysfunction induced by laparotomy during the whole postoperative period. Our findings provided information for studying the relationship of systemic inflammation and neuroinflammation by using one single animal model.

Contradictory studies showed that anesthesia improved spatial memory in young rats [24] or prevented against organ protection or cytoprotective effect by attenuating systemic or local inflammatory responses and apoptosis after ischemia-reperfusion injury [25, 26] or sepsis [27]. Sevoflurane has minimal impact on cytokine and microglia activity [28, 29]. In this study, significant decreases in MCP-1 and Iba1^+^ microglia after brief exposure to sevoflurane may suggest its suppressive effects on cytokine production as well as glial activation.

Beyond its classical role in stabilizing microtubule, tau has other cellular functions such as regulating microtubules assembly, dynamic behavior, and the axonal transport under physiological conditions via phosphorylation [30, 31]. Accumulation of abnormally phosphorylated tau is a major neuropathological feature of tauopathies in neurodegenerative disorders [9]. In tauopathies, the intracellular soluble tau forms filamentous structures of aggregated, hyperphosphorylated tau, which are associated with synaptic loss and neuronal death. Therefore, based on their role in the pathogenesis of cognitive impairment and neuronal apoptosis, tau protein characteristics may have diagnostic and possibly predictive implications in postoperative cognitive changes [18]. Furthermore, the ability of tau protein transferring between neurons trans-neuronally and trans-synaptically via the extracellular space [32] may contribute to the toxic relationship between tau oligomers and inflammation. Tau protein could spread and initiate a feed-forward cycle to magnify inflammation even though the inflammation occurred in disease prior to the formation of larger aggregates [33]. In our study, the significant elevation of tau phosphorylation in the frontal cortex and hippocampus (S404, AT8, and AT180) on postoperative day 14 may characterize the pathological profiles of cognitive impairment after laparotomy.

A growing body of evidence supports the critical activation by multiple cytokines of the Janus kinase (JAK)/signal transducer and activator of transcription (STAT) pathway, the Ras-Raf-mitogen-activated protein kinase (MAPK) pathway, in the pathogenesis of various neuro-inflammatory and neurodegenerative disorders of the CNS [34, 35]. The JAK/STAT pathway is involved in many cellular processes, including cell growth and differentiation, immune functions, and synaptic plasticity [36]. In addition, the JAK/STAT pathway may also have a role in memory formation [37] through either modulating the microtubule stability [38] or regulating the synaptic plasticity [36]. As a key effector of neuronal survival after injury, STAT3 influences neuronal survival during development, gliogenesis regulation, neuroinflammation, and neurodegeneration [35, 39, 40]. Therefore, the upregulation of STAT3 phosphorylation by laparotomy may contribute to the increases in the population of GFAP^+^ astrocytes and Iba1^+^ microglia in the frontal cortex and hippocampus, which were then attenuated by ibuprofen application. The involvement of the MAPK pathway and GSK3β for the tau-dependent neurotoxicity was addressed to dissect the mechanism concerning cognitive dysfunction resulted from laparotomy [34]. The inhibition of phosphatase activity negatively modulated neuronal tau phosphorylation, which might be the major signal transduction target of laparotomy. The relative increase of Ser9 epitope of GSK3β after ibuprofen consumption, coupled with the decrease of PP2A, an important tau phosphatase, suggests that GSK3B plays a relatively more important role in the effect of ibuprofen on tau phosphorylation.

The attenuation of the pro-inflammatory response is known to be beneficial for functional recovery after CNS injuries, and the inhibition of systemic inflammation prevented the changes demonstrated in this study. Ibuprofen is a widely used non-steroidal anti-inflammatory drug (NSAID). Our study confirmed that sustained administration of ibuprofen prevented cognitive deficits correlated with a reduction in tau phosphorylation following laparotomy [41]. During this process, ibuprofen suppressed the activation of microglia and reactive astrocytes, as well as the pro-inflammatory cytokines. The major contributor to all these changes may be the attenuation of stress signaling pathways by ibuprofen. Specifically, ibuprofen consistently prevented the activation of JAK/STAT signaling pathway and JNK. For tau phosphorylation, the stress signaling pathways and major tau kinase GSK3β may be the major mechanisms responsible for the attenuating effect of ibuprofen against tau phosphorylation following laparotomy. The cell survival-related kinases and tau phosphatase were relatively less prominent.

In order to demonstrate and integrate the range of changes in a single model, we used an approach of modulating systemic inflammation with the non-steroidal anti-inflammatory agent ibuprofen. While we demonstrated that the approach of giving the drug for the entirety of the experimental period brought benefits, we have not explored whether just dampening the initial inflammatory response with a shorter course would produce a similar response. This question is of clinical significance as prolonged non-steroidal use, especially in the perioperative period, may cause an unfavorable risk-benefit ratio, particularly in the elderly population. Taken together, we have shown that neuroinflammation may be protracted after surgery, and this causes adverse changes in the brain, the effects of which can be attenuated by the use of anti-inflammatory treatment.

## Conclusions

In summary, we established a stable and sensitive animal model for investigating neuropathological variants induced by systemic inflammation, in which several key components contributing to the development of cognitive dysfunction have been demonstrated comprehensively, such as inflammation, tauopathy, and gliosis. And the systemic and neural inflammation profiles were further described during the cognitive decline processes.

## Additional file

Additional file 1: **Table S1.** Analgesia measurement by von Frey filament at postoperative 24 h. **Figure S1.** Activation of microglia in the brain from surgical mice. Representative confocal microphotographs presented the activation of Iba1^+^ microglia in the frontal cortex (M2 region, top boxes; sensory cortex, bottom boxes) and hippocampus induced by laparotomy. **Figure S2.** Activation of astrocyte in the frontal cortex from surgical mice. (**a**) Representative confocal microphotographs presented the activation of GFAP^+^ astrocyte in the motor cortex (M2: secondary motor cortex; AIV: agranular insular cortex, ventral part) on POD 14. (**b**) Representative confocal microphotographs presented the activation of GFAP^+^ astrocyte in the sensory cortex (aci: anterior commissure intrabulbar part; OV: olfactory ventricle (olfactory part of lateral ventricle)) induced by laparotomy on POD 14. (ZIP 27129 kb)

## Abbreviations

AD: Alzheimer’s disease
CNS: Central nervous system
CULATR: Committee on the Use of Live Animals in Teaching and Research
DAPI: 4′,6-Diamidino-2-phenylindole
DG: Dentate gyrus
ERK: Extracellular signal-regulated kinases
GAPDH: Glyceraldehyde 3-phosphate dehydrogenase
GFAP: Gilial fibrillary acidic protein
GSK3β: Glycogen synthase kinase-3β
IL: Interleukin
IL-1β: Interleukin-1-β
JAK: Janus kinase
JNK: Stress-activated protein kinases (SAPK)/c-Jun N-terminal kinase
LAU: Laboratory Animal Unit
LPS: Lipopolysaccharide
MAPK: Mitogen-activated protein kinase
MCP-1: Monocyte chemoattractant protein-1
NSAID: Non-steroidal anti-inflammatory drug
OFT: Open field test
POCD: Postoperative cognitive dysfunction
POD: Postoperative day
PP2A: Protein phosphatase 2
STAT: Signal transducer and activator of transcription
TNF-α: Tumor necrosis factor-α
