# Supplementary material for: Evidence of the impact of systemic inflammation on neuroinflammation from a non-bacterial endotoxin animal model
Source: J Neuroinflammation. 2018 May 17;15:147. doi: 10.1186/s12974-018-1163-z (PMC5960121; doi:10.1186/s12974-018-1163-z)
Supplement: Supplementary file 1 — Table S1. Analgesia measurement by von Frey filament at postoperative 24 h. Figure S1. Activation of microglia in the brain from surgical mice. Representative confocal microphotographs presented the activation of Iba1+ microglia in the frontal cortex (M2 region, top boxes; sensory cortex, bottom boxes) and hippocampus induced by laparotomy. Figure S2. Activation of astrocyte in the frontal cortex from surgical mice. (a) Representative confocal microphotographs presented the activation of GFAP+ astrocyte in the motor cortex (M2: secondary motor cortex; AIV: agranular insular cortex, ventral part) on POD 14. (b) Representative confocal microphotographs presented the activation of GFAP+ astrocyte in the sensory cortex (aci: anterior commissure intrabulbar part; OV: olfactory ventricle (olfactory part of lateral ventricle)) induced by laparotomy on POD 14. (ZIP 27129 kb) [file 12974_2018_1163_MOESM1_ESM.zip › Supplementary Data for JNI-2018-4-14.docx]

**Evidence of the impact of systemic inflammation on neuroinflammation from a non-bacterial endotoxin animal model**

**Supplementary Data**

**Supplementary Table 1.** Analgesia measurement by Von Frey filament at postoperative 24 hours

| CON | SEVO | LAP |
| --- | --- | --- |
| 3.51 ± 0.27 | 3.50 ± 0.17 | 3.58 ± 0.17 |

Data of VFF value (g/f) were presented as Mean±SD.

**

**

**Supplementary Fig. 1** Activation of microglia in the brain from surgical mice. Representative confocal microphotographs presented the activation of Iba1^+^ microglia in the frontal cortex (M2 region, top boxes; sensorycortex, bottom boxes) and hippocampus induced by laparotomy.





**Supplementary Fig. 2** Activation of astrocyte in the frontal cortex from surgical mice. (**a**) Representative confocal microphotographs presented the activation of GFAP^+^ astrocyte in the motor cortex (M2: secondary motor cortex; AIV: agranular insular cortex, ventral part) on POD 14. (**b**) Representative confocal microphotographs presented the activation of GFAP^+^ astrocyte in the sensory cortex (aci: anterior commissure intrabulbar part; OV: olfactory ventricle (olfactory part of lateral ventricle)) induced by laparotomy on POD 14.
